# Constitutively expressed Protocadherin-α regulates the coalescence and elimination of homotypic olfactory axons through its cytoplasmic region

**DOI:** 10.3389/fnmol.2012.00097

**Published:** 2012-10-16

**Authors:** Sonoko Hasegawa, Takahiro Hirabayashi, Takahiko Kondo, Ken Inoue, Shigeyuki Esumi, Atsushi Okayama, Shun Hamada, Takeshi Yagi

**Affiliations:** ^1^KOKORO-Biology Group and CREST-JST, Laboratories for Integrated Biology, Graduate School of Frontier Biosciences, Osaka UniversityOsaka, Japan; ^2^Department of Morphological Neural Science, Graduate School of Medical Sciences, Kumamoto UniversityKumamoto, Japan; ^3^Department of Nutrition and Health Sciences, Fukuoka Women's UniversityFukuoka, Japan

**Keywords:** olfactory, axon, protocadherin, Pcdh, convergence, elimination, neural circuit, neuron

## Abstract

Olfactory sensory neuron (OSN) axons coalesce into specific glomeruli in the olfactory bulb (OB) according to their odorant receptor (OR) expression. Several guidance molecules enhance the coalescence of homotypic OSN projections, in an OR-specific- and neural-activity-dependent manner. However, the mechanism by which homotypic OSN axons are organized into glomeruli is unsolved. We previously reported that the clustered protocadherin-α (Pcdh-α) family of diverse cadherin-related molecules plays roles in the coalescence and elimination of homotypic OSN axons throughout development. Here we showed that the elimination of small ectopic homotypic glomeruli required the constitutive expression of a Pcdh-α isoform and Pcdh-α's cytoplasmic region, but not OR specificity or neural activity. These results suggest that Pcdh-α proteins provide a cytoplasmic signal to regulate repulsive activity for homotypic OSN axons independently of OR expression and neural activity. The counterbalancing effect of Pcdh-α proteins for the axonal coalescence mechanisms mediated by other olfactory guidance molecules indicate a possible mechanism for the organization of homotypic OSN axons into glomeruli during development.

## Introduction

The olfactory system can recognize and discriminate an enormous number of odor molecules in the external environment. In this system, individual olfactory sensory neurons (OSNs) in the olfactory epithelium (OE) express only one type of functional odorant receptor (OR) from ~1000 genes in mice (Buck and Axel, 1991; Chess et al., 1994; Malnic et al., 1999). The OSNs expressing one type of OR project and coalesce their axons into specific glomeruli that are spatially arranged on the surface of the olfactory bulb (OB) (Ressler et al., 1994; Vassar et al., 1994; Mombaerts et al., 1996).

The coalescence of OSN axons into glomeruli is directed by the expressed OR and is dependent on neural activity (Feinstein and Mombaerts, 2004; Feinstein et al., 2004; Mombaerts, 2006; Serizawa et al., 2006). In this process, guidance molecules including cell adhesion molecules and receptors organize the OSN axon projections; these guidance molecules include ephrin/Eph, Semaphorin/neuropilin, Plexin, BIG-2, and Kirrel2/3 (Schwarting et al., 2000, 2004; Walz et al., 2002; Cutforth et al., 2003; Taniguchi et al., 2003; Imai et al., 2006; Serizawa et al., 2006; Col et al., 2007; Kaneko-Goto et al., 2008; Takeuchi et al., 2010). The expression levels of these guidance molecules in the OSNs are regulated by OR-derived signals and the anatomical location of the OSNs in the OE, resulting in the coalescence of homotypic OSNs. Thus, the distinct expression levels and combinations of guidance molecules in the OSNs constitute one basic mechanism for the approximate projection and coalescence of OSN axons.

The protocadherin-α (*Pcdh*-α) genes belong to the clustered Pcdh families, which encode cadherin-related proteins with distinct domains derived from multiple variable exons: six extracellular cadherin domains, a transmembrane domain, and a short cytoplasmic domain (Kohmura et al., 1998; Wu and Maniatis, 1999; Yagi, 2012). They also have a common cytoplasmic tail (type A or B) derived from three or four constant exons (Kohmura et al., 1998; Sugino et al., 2000). The diverse Pcdh-α molecules are required for the coalescence of homotypic OSN axons into the OR-specific glomeruli of the OB (Hasegawa et al., 2008).

To address the functional significance of the diversity of Pcdh-α proteins, here we produced mutants in which exons α*2*–α*C2*, i.e., all but exon α*1*, were deleted in the variable region of the *Pcdh*-α cluster. Surprisingly, in the mutant mice, the remaining α1 isoform compensated for the others, and was constitutively expressed in the neurons including OSNs. The coalescence of OSN projections in these mutants looked normal. In contrast, loss of the common cytoplasmic region from the Pcdh-α proteins disrupted the axonal coalescence. These findings indicate that constitutively expressed Pcdh-α proteins provide a cytoplasmic signal to regulate axonal coalescence and eliminate ectopic glomeruli. We also showed that the expression and function of Pcdh-α were not dependent on either OR-specific signaling or OSN-derived neural activity. Based on these results, we propose that Pcdh-α has an activity that causes repulsion (or elimination) in homotypic OSN axons, that is different from the coalescence activity provided by other olfactory guidance molecules.

## Materials and methods

### Animal experiments

All the experimental procedures were in accordance with the Guide for the Care and Use of Laboratory Animals of the Science Council of Japan and were approved by the Animal Experiment Committee of Osaka University.

### Generation of Pcdh-α mutant mice

Similar to produce *Pcdha*^Δ*CR*/Δ*CR*^ mice (Hasegawa et al., 2008), we generated *Pcdha*^Δ*CR*2/Δ*CR*2^ mice using the CAG-Cre transgene and two targeted mutations: one G1loxP allele and another Δ*A* allele (Katori et al., 2009). The *Pcdha*^Δ*CR*2/Δ*CR*2^ mice expressed no Pcdh-α protein similar to *Pcdha*^Δ*CR*/Δ*CR*^ mice (data not shown).

By mating G16Neo mutant mice, in which two *loxP* sites were inserted between exons α*1* and α*2* (Noguchi et al., 2009), G1loxP mice in which a *loxP* site was inserted between exons α*c2* and α*CR1* (Hasegawa et al., 2008), and synaptosomal complex protein1 (Sycp)-Cre transgenic mice (Noguchi et al., 2009), male mice carrying the G16Neo allele, G1loxP allele, and Sycp-Cre transgene were generated. These mice were crossed with C57BL/6 females, and the genotypes of pups were determined by Southern blotting using probes amplified by PCR with Probe-G16-F (5′-GGAGGACATGCACAAGTCATG-3′) and Probe-G16-R (5′-TTGTGGTGTACAGCGACACC-3′) primers, and by PCR using G16 primer (5′-GGCTATCCTGTGCTACAGAAC-3′), G16-GTP-R2 primer (5′-CCAATTAATATTTGAGATTCATCCCC-3′), and G1-GTP-R primer (5′- GCCCAGGATGGCTCAAATTC-3′). Some pups carried the *Pcdha*^Δ(*2–c2*)^ or the *Pcdha*^*dup*(*2–c2*)^ allele generated by trans-allelic targeted meiotic recombination (TAMERE) in the testis (Herault et al., 1998) (Figure 5). *Pcdha*^Δ*A*/Δ*A*^ mutant mice with a truncated Pcdh-α protein were described previously (Katori et al., 2009).

### RT-PCR

Total RNA from mouse whole brain was extracted with TRIzol Reagent (Life Technologies), and the cDNAs were synthesized using SuperScriptIII reverse transcriptase (Life Technologies), according to the manufacturer's protocol. Forward primers were α*1*, 5′-GTGACCACGCAGAAGTAAAT-3′; α*2*, 5′-GAAGAGAGACAACCACCCTT-3′; α*3*, 5′-GACAAACTGGTTGGAGACAT-3′; α*4*, 5′-CAATTGCAGTCTGCAGAGGA-3′; α*5*, 5′-ACCTCAGGGACCCAGCTCTA-3′; α*6*, 5′-GCATCAGGATTTGAACGACG-3′; α*7*, 5′-CCTACCTCAGGGTCCCAGCT-3′; α*8*, 5′-CCATCTGTTTCTTTGGACTC-3′; α*9*, 5′-GGAAAGTCATTCTGTTGGAG-3′; α*10*, 5′-GGTTCTGGAGATAGTGGAGT-3′; α*11*, 5′-GGAAAGACAGGAGTCAGAGT-3′; α*12*, 5′-GTCAGAGAGAAAGGCAGGTA-3′; α*c1*, 5′-GGGGATCATTCAAATGTGGA-3′; α*c2*, 5′-CCGGGAACCTGATTATCCTA-3′, and reverse primer was α*CR*5′-GACTGTTTGGGGTTGCC-3′. Quantitative PCR analysis was performed with the SYBR Green Master Mix (Life Technologies) using ABI 7900HT (Life Technologies) with primers for α*1*, 5′-CCCAGGTTTGAACATAGGC-3′ and 5′-CGAGGCAGAGTAGCGCC-3′; for α*CR*, 5′-AGAGCAGGCATGCACAGC-3′ and 5′-GACTGTTTGGGGTTGCC-3′; *GAPDH*, 5′-GACTTCAACAGCAACTCCCAC-3′ and 5′-TCCACCACCCTGTTGCTGTAG-3′. For statistical analysis of the qPCR data, One-Way ANOVA, Tukey's test was used.

### Single cell RT-PCR

Single cell RT-PCR analysis of OSNs was performed as described previously (Esumi et al., 2005). Briefly, single OSNs were obtained from the OE of adult OMP-GFP mice (a generous gift from Dr. Mombaerts). We picked up single GFP-positive OSNs with a glass capillary and placed them in individual PCR tubes. After cDNA synthesis from the single cells, the first multiplex PCR was performed using a 5′ PCR primer designed to contain a consensus sequence common to all the variable exons of the *Pcdh*-α family. Next, we amplified each *Pcdh*-α isoform using a specific primer pair and semi-nested second PCR. Finally, we directly sequenced the PCR-amplified products to identify which *Pcdh*-α mRNAs were expressed in individual cells.

### Immunoblot analysis

Mouse whole brains were homogenized in 0.32 M sucrose containing 1 mM EDTA and protease inhibitors, with a Dounce homogenizer. The homogenates were spun at 800 × g for 10 min. The supernatants were spun at 20,000 × g at 4°C for 30 min. The pellets were lysed with lysis buffer (20 mM Tris-HCl, pH 7.5, 150 mM NaCl, 1 mM EDTA, 1% Triton X-100, protease inhibitors) and spun at 20,000 × g at 4°C for 30 min. The supernatants were subjected to SDS-PAGE followed by immunoblot analysis using a rabbit anti-Pcdha antibody (Murata et al., 2004).

### *In situ* hybridization histochemistry

*In situ* hybridization histochemistry was performed as described previously (Katori et al., 2009; Noguchi et al., 2009). Briefly, 10 μm-thick fresh-frozen sections were prepared and hybridized with a constant-region cRNA probe for α*4* cDNA (nucleotides 2518–4559) to detect all the *Pcdh*-α mRNA members. To detect certain variable isoforms of the *Pcdh*-α mRNAs, probes for α*1* cDNA (nucleotides 396–1134), and α*11* cDNA (nucleotides 398–2367) were used.

### Immunohistochemistry

Immunohistochemistry was performed as described previously (Hasegawa et al., 2008). We used antibodies against mOR-EG and MOR28, kindly given by Dr. Yoshihara, and a rabbit anti-Pcdhα CR antibody, a generous gift from Dr. Watanabe.

### β-galactosidase histochemistry

β-galactosidase histochemistry was performed as described previously (Hasegawa et al., 2008). Littermates from *Pcdha*^+/Δ*CR*^, *Pcdha*^+/Δ*CR*2^, *Pcdha*^+/Δ(*2–c2*)^, and *Pcdha*^+/Δ*A*^ heterozygous parents were examined to compare WT and mutant mice that were also homozygous for M71-IRES-taulacZ, MOR23-IRES-taulacZ, or P2-IRES-taulacZ (kindly provided from Dr. Mombaerts). For this study, we mainly used *Pcdha*^Δ*CR*/Δ*CR*^ mice, except for counting the P2 glomeruli at P7 and P30, for which we used *Pcdha*^Δ*CR*2/Δ*CR*2^ mice. The phenotypes described in this study were similar for these strains (see Table 1).

**Table 1:** **Number of glomeruli per half-bulb in sectional analysis**.

	**Age**	**(*n*)**	**Lateral**	**(Min–Max)**	**(*n*)**	**Medial**	**(Min–Max)**
**10 μM-THICK SECTION**							
mOR-EG	P30						
WT		(10)	1.0	( – )	(12)	1.0	(1.0–3.0)
ΔCR/ΔCR		(10)	1.6	(1.0–4.0) *P* = 0.0301	(09)	1.4	(1.0–2.0) *P* = 0.0139
MOR28	P30						
WT		(10)	1.2	(1.0–2.0)	(10)	1.0	( – )
ΔCR/ΔCR		(10)	2.4	(1.0–4.0) *P* = 0.0053	(10)	1.3	(1.0–3.0) *P* = 0.1468
**50 μM-THICK SECTION**							
P2	P30						
WT		(16)	1.9	(1.0–3.0)	(17)	2.1	(1.0–4.0)
ΔCR/ΔCR		(20)	4.0	(3.0–6.0) *P* < 0.0001	(20)	4.1	(2.0–6.0) *P* < 0.0001
P2	P30						
WT		(13)	2.7	(2.0–4.0)	(14)	2.2	(1.0–4.0)
ΔCR2/ΔCR2		(09)	5.3	(5.0–6.0) *P* < 0.0001	(09)	3.8	(2.0–6.0) *P* = 0.0036
MOR23	P30						
WT		(12)	1.3	(1.0–2.0)	(12)	1.0	( – )
ΔCR2/ΔCR2		(12)	2.0	(1.0–3.0) *P* = 0.0099	(12)	1.6	(1.0–3.0) *P* = 0.0059

n = half-bulb, Mann–Whitney U-test.

### Unilateral naris occlusion

Unilateral naris occlusion was performed as described previously (Philpot et al., 1997).

### Image analysis

Fluorescent digital images were captured using a fluorescence microscope (Olympus BX51) equipped with a DP50 CCD camera (Olympus).

### Statistical analysis

Statistical analysis was conducted using StatView J-4.5 (SAS Institute, Cary, NC). Comparison of the number of glomeruli was performed by the Mann–Whitney test. Values in graphs were expressed as the mean ± SEM.

## Results

### Abnormal axonal coalescence of the OSNs in *Pcdh*-α mutants

**Figure 1:**
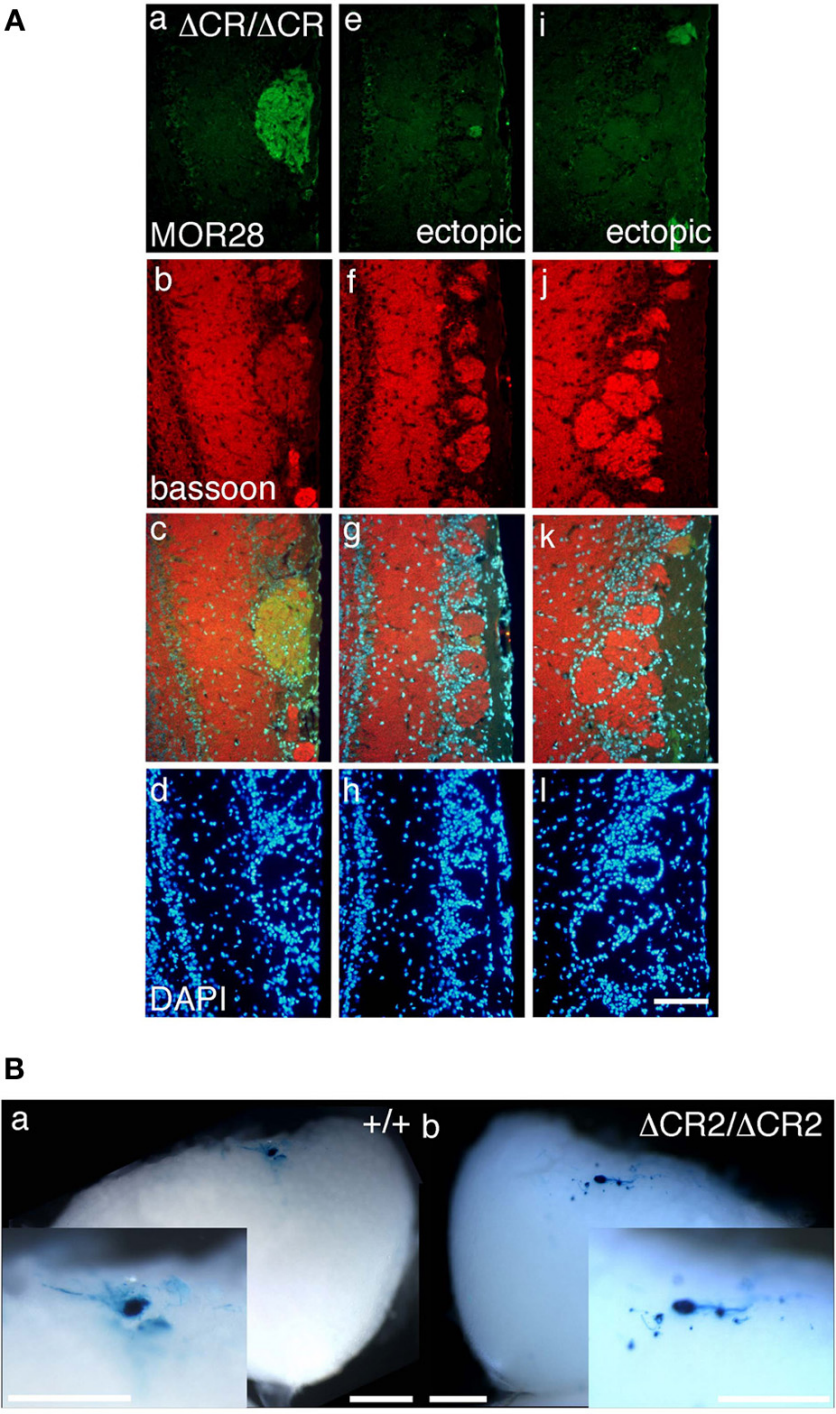
**Ectopic glomeruli in MOR28 and P2 OSN projections. (A)** Representative examples of the lateral side of three MOR28 glomeruli, (a: one innate glomerulus, e and i: two small ectopic glomeruli). (b, f, and j) Immunoreactivity for bassoon (a presynaptic active-zone protein) was observed in all the MOR28 glomeruli in the *Pcdha*^Δ*CR*/Δ*CR*^ mice. (c, g, and k) merged images. (d, h, and l) DAPI. Scale bar, 100 μm. **(B)** Lateral P2 glomeruli in whole-mount OBs from WT (a) and *Pcdha*^Δ*CR*2/Δ*CR*2^ (b) mice at P7. In the *Pcdha*^Δ*CR*2/Δ*CR*2^ mice, small ectopic glomeruli were observed near the large main glomerulus. Scale bars, 500 μm.

The diverse *Pcdh*-α family is required for the normal coalescence of OSN axons into glomeruli and for the elimination of ectopic glomeruli in the OB. In *Pcdh*-α-deficient mice, abnormal, small ectopic glomeruli are observed for OSNs expressing the M71, M72, and MOR23 ORs (Hasegawa et al., 2008). To confirm whether Pcdh-α is involved in the axonal coalescence of homotypic OSNs in the glomeruli in ventral and dorsal positions of the OB, we examined the axonal coalescence of OSNs expressing the ORs MOR28 (ventral side) and mOR-EG (dorsal side). The glomerular position of MOR28, but not of mOR-EG, is greatly influenced by the disruption of *Neuropilin-2* or *Plexin-A3* (Takeuchi et al., 2010). The disruption of *BIG-2* impairs the axonal coalescence of MOR28 and mOR-EG OSNs to different degrees (Kaneko-Goto et al., 2008). Immunostaining with OR-specific antibodies showed increased numbers of glomeruli in the OSNs at P30 in the *Pcdh*-α-deficient (*Pcdha*^Δ*CR*/Δ*CR*^) vs. wild-type (WT) mice, for mOR-EG (averages, lateral 1.6 vs. 1.0; medial 1.4 vs. 1.0, respectively) and MOR28 (lateral 2.4 vs. 1.2; medial 1.3 vs. 1.0) (Table 1, Figure 1A), similar to the previous results for M71, M72, and MOR23 (Hasegawa et al., 2008).

The organization and projections of axons expressing the OR P2 are well-studied; P2 OSNs possess distinct characteristics from other OR-expressing OSNs, such as a lower sensitivity to the loss of neural activity (Lin et al., 2000; Zheng et al., 2000) and a distinct zonal distribution within the OE. Therefore, we next examined the P2 glomeruli in WT and *Pcdh*-α-deficient (*Pcdha*^Δ*CR*/Δ*CR*^ and *Pcdha*^Δ*CR*2/Δ*CR*2^) mice with a P2-IRES-taulacZ locus. As seen for the other ORs, the number of P2 glomeruli increased in the *Pcdh*-α-deficient mice (Figure 1B). In whole-mount preparations, the lateral half-bulb showed a mean of 1.9 labeled glomeruli in WT mice (*n* = 16 half-bulbs), and 4.0 glomeruli in the *Pcdh*-α-deficient mice (*n* = 20 half-bulbs). In the medial half-bulb, there were 2.1 glomeruli in the WT (*n* = 17 half-bulbs), and 4.1 glomeruli in the *Pcdh*-α-deficient mice (*n* = 20 half-bulbs) (Table 1). Thus, all the homotypic OSNs examined showed a similar phenotype of increased ectopic glomeruli in *Pcdh*-α-deficient mice. These results suggested that Pcdh-α's function contributes to axonal coalescence and the elimination of ectopic glomeruli for all kinds of homotypic OSNs.

### Glomerular formation in *Pcdh*-α mutant mice at late-embryonic and neonatal stages

**Figure 2:**
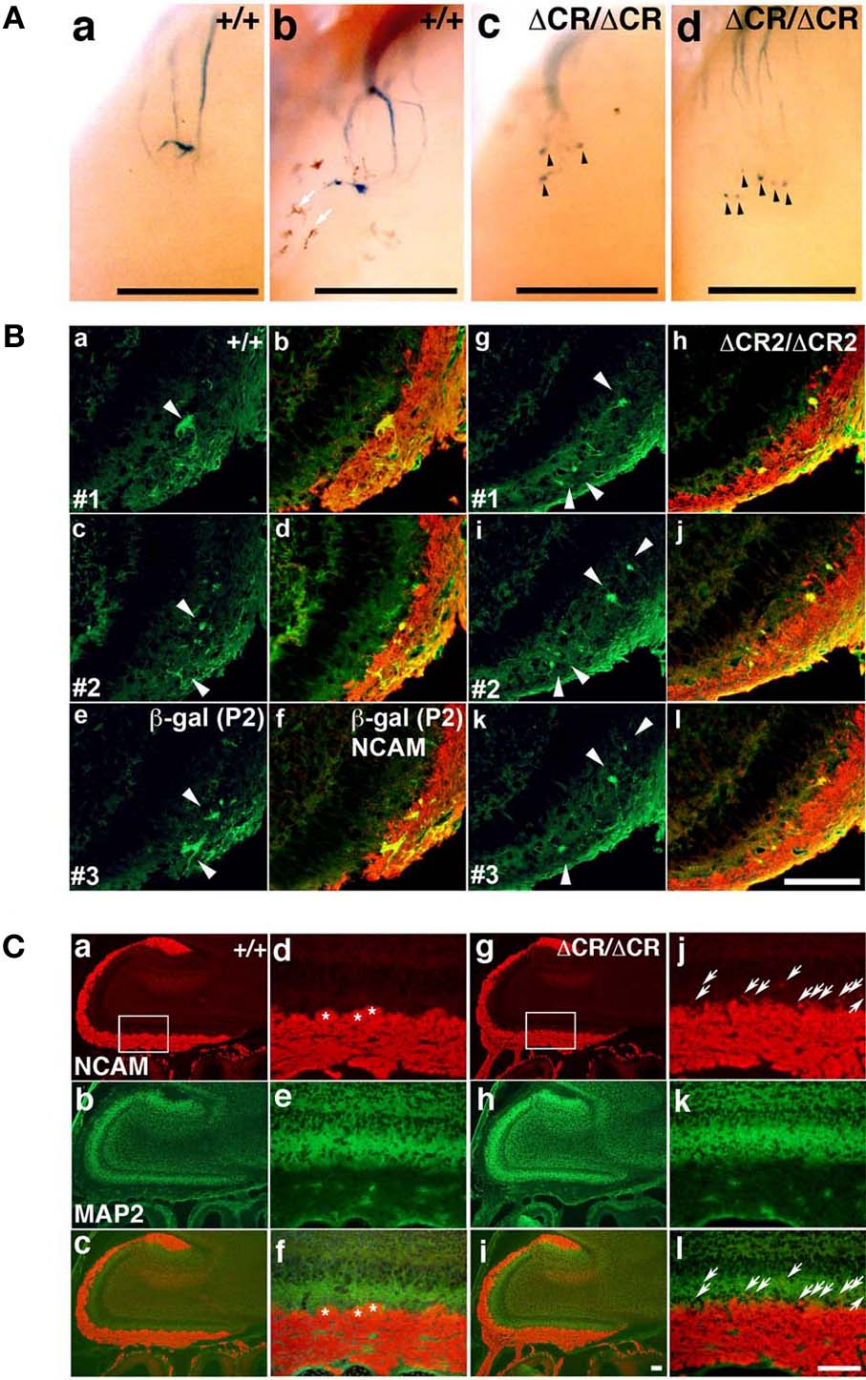
**Abnormal glomerular morphology in prenatal and neonatal *Pcdha*^Δ*CR*/Δ*CR*^ mice. (A)** Ectopic glomerulus-like structures of M71-expressing OSNs were found in neonatal *Pcdha*^Δ*CR*/Δ*CR*^ mice by whole-mount observation. X-gal-stained lateral M71 glomeruli in whole-mounted OBs from WT (a, b) or *Pcdha*^Δ*CR*/Δ*CR*^(Δ*CR*/Δ*CR*) (c, d) mice at P0. In *Pcdha*^Δ*CR*/Δ*CR*^ mice, abnormal axonal projections from the olfactory nerve were often detected (arrowheads). Melanocytes (arrows) were visible on some of the whole-mount preparations of the olfactory bulbs. Scale bars, 500 μm. **(B)** Sectional analysis of the coalescence of WT (a–f) and *Pcdha*^Δ*CR*2/Δ*CR*2^ (g–l) P2 axons on embryonic day (E) 17.5. Serial sections of OBs were double-labeled with anti-β-galactosidase (for P2, green) and anti-NCAM (red) antibodies. There were more P2 glomerulus-like structures (arrowheads) in the *Pcdha*^Δ*CR*2/Δ*CR*2^ (Δ*CR*2/Δ*CR*2) mice (See Figure 3A). Scale bar, 100 μm. **(C)** OBs in WT (a–c) and *Pcdha*^Δ*CR*/Δ*CR*^ mice (g–i) were double-labeled with anti-NCAM (red) and anti-MAP2 (green) antibodies. Due to the orientation shown in panels (d) and (j), in *Pcdha*^Δ*CR*/Δ*CR*^ mice, the OSN axons appeared to extend beyond the normal confines of the glomerular layer and often terminated as an intensely stained spatially restricted and condensed structure (j and l, arrows). In addition, the primary axons terminated in less clearly defined glomeruli in *Pcdha*^Δ*CR*/Δ*CR*^ (Δ*CR*/Δ*CR*) than in WT (+/+) mice. Primary glomerular structures could be detected in WT (d, f, asterisks) but not *Pcdha*^Δ*CR*/Δ*CR*^ mice (j, l) (See Figures 6B,C). Immunostaining with anti-MAP2 (green) antibody did not show significant differences between WT (e) and *Pcdha*^Δ*CR*/Δ*CR*^ (k) mice. Scale bar, 100 μm.

**Figure 3:**
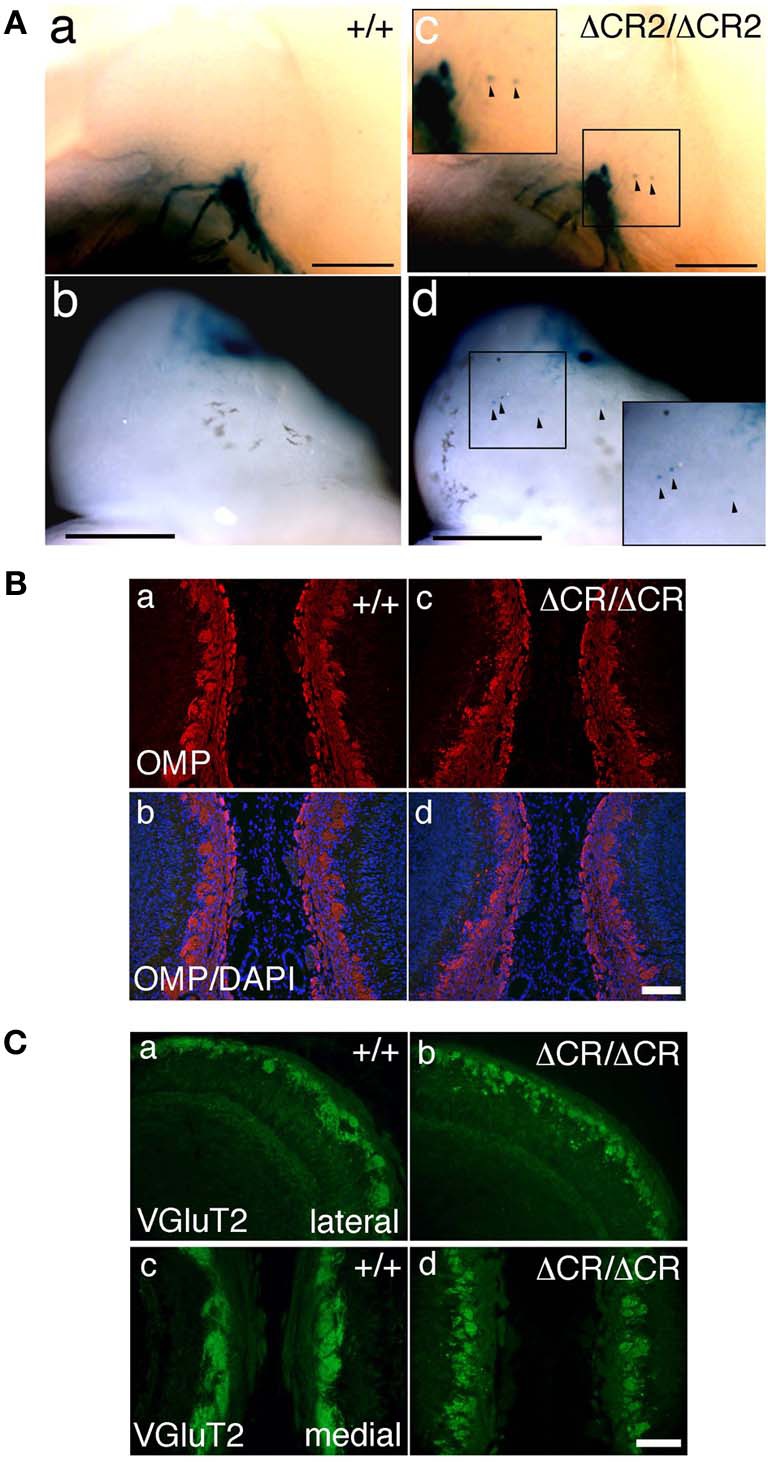
**Ectopic glomerulus-like structures of P2-expressing OSNs in neonatal *Pcdha*^Δ*CR*2/Δ*CR*2^ mice found by whole-mount observation. (A)** X-gal-stained P2 glomeruli in the P0 OBs of WT (a, b) or *Pcdha*^Δ*CR*2/Δ*CR*2^ (c, d) mice. In *Pcdha*^Δ*CR*2/Δ*CR*2^ mice, abnormal axonal projections from the olfactory nerve were often detected (arrowheads). Scale bars, 500 μm. **(B)** Double staining with an anti-OMP antibody and DAPI in the P0 OBs in coronal sections of WT (a, b) or *Pcdha*^Δ*CR*/Δ*CR*^ (c, d) mice. Scale bar, 100 μm. **(C)** Immunostaining with an anti-VGluT2 antibody in the lateral (a, b) and medial (c, d) sides of the P0 OBs in WT (a, c) or *Pcdha*^Δ*CR*/Δ*CR*^ (b, d) mice. Scale bar, 100 μm.

As described above, the *Pcdh*-α-deficient mice had multiple, small, extraneous glomeruli for all the OSN axons examined. These ectopic glomeruli persist until adulthood (Hasegawa et al., 2008). To determine whether the abnormalities in the *Pcdh*-α-deficient olfactory system resulted primarily from an inability of homotypic OSN axons to coalesce, we next analyzed the formation of glomeruli during early development using mice from crosses between M71-IRES-taulacZ or P2-IRES-taulacZ and WT or *Pcdha*^Δ*CR*/Δ*CR*^ mice (Hasegawa et al., 2008). The glomerular structures are initiated on embryonic day (E) 15–16, when both OSN fibers and mitral cell dendrites contribute to the formation of glomerulus-like structures (Blanchart et al., 2006). In the *Pcdha*^Δ*CR*/Δ*CR*^ mice, we observed some stray marked fibers that projected to inappropriate regions distant from the target site, and many more M71 and P2 glomerulus-like structures than in WT mice, even on postnatal day 0 (P0) (arrowheads in Figures 2A, 3A).

Sectional analysis of the OBs on E17.5 clearly showed the abnormal coalescence of P2 axons—multiple small and extraneous P2 glomerular-like structures—during the early formation of glomeruli (Figure 2B). These results indicated that the ectopic glomeruli found in the *Pcdha*^Δ*CR*/Δ*CR*^ mice, which were often maintained until adulthood, were derived from the late-embryonic stage before birth, suggesting that Pcdh-α is involved in the initial axonal coalescence of the homotypic OSNs as well as the elimination of ectopic glomeruli during postnatal development. Ebrahimi and Chess (2000) showed that the successful coalescence of OSN axons at adulthood depends on the population size of OSNs (more OSNs result in better coalescence). On the other hand, small ectopic glomeruli that appear in normal newborn mice completely disappear by adulthood.

To analyze the dendrites of bulb neurons and OSN axons in more detail, we performed immunostaining for NCAM, an axonal marker and for MAP-2, a dendritic marker. In WT mice at P0, the outline of individual glomeruli surrounded by periglomerular cells could be recognized in the glomerular layer by NCAM staining (asterisks in Figure 2C d, f). In contrast, such glomerular structures were not seen in *Pcdha*^Δ*CR*/Δ*CR*^ mice, in which NCAM-positive axons from the OSNs were often found in the external plexiform layer (arrows in Figure 2C j, l). These abnormal axonal clusters were confirmed by immunostaining with anti-OMP, a marker of OSNs and with anti-VGluT2, a presynaptic marker (Figures 3B,C). These results suggested that abnormal axonal coalescence was a common feature of all the homotypic ONSs in the *Pcdha*^Δ*CR*/Δ*CR*^ mice. These observations support the idea that Pcdh-α helps to determine the precise organization of homotypic OSN axon projections during all developmental stages.

### Differential expression of each *Pcdh*-α isoform in single neurons including OSNs

**Figure 4:**
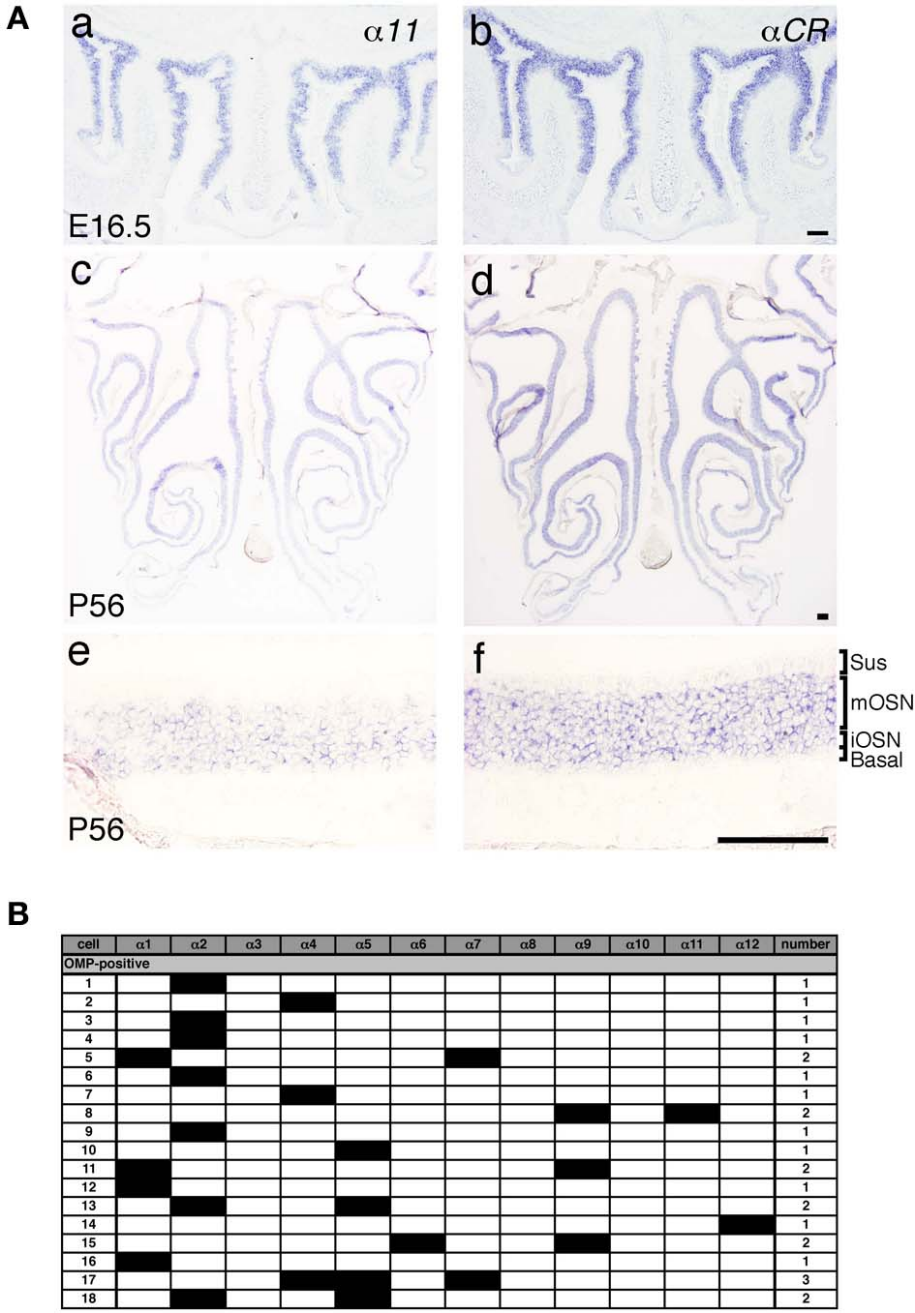
**Expression of *Pcdh*-α mRNAs in OSNs of the OE. (A)** *In situ* hybridization histochemistry with an α*11* isoform-specific probe and a α*CR* probe for all the *Pcdh*-α isoforms at E16.5 (a, b) and P56 (c–f). High magnifications are shown in (e) and (f). Sus, sustentacular cell-body layer; mOSN, mature OSN cell-body layer; iOSN, immature OSN cell-body layer; Basal, basal cell body layer. Scale bars, 100 μm. **(B)** Single cell RT-PCR analysis of individual OSNs using adult OMP-GFP mice.

*Pcdh*-α mRNAs are extensively expressed in almost all of the OSNs from E11.5 to adulthood, and Pcdh-α proteins are enriched in the OSN axons and their terminals in the glomeruli (Hasegawa et al., 2008). However, the mechanisms by which the diverse Pcdh-α family contributes to the axonal coalescence into glomeruli remain unclear. To address this issue, we examined the expression pattern of various *Pcdh*-α isoforms in the OSNs. First, *in situ* hybridization histochemistry with probes for isoform-specific α*11* and isoform-common α*CR* was performed for the OSNs of the OE. Extensive staining was observed at E16.5 for both the α*11* and α*CR* probes (Figure 4A a, b). At P56, their expressions were widely observed in the OE, and appeared in both the immature and mature OSN cell body layers but not in the sustentacular or basal cell layers (Figure 4A c, d). In contrast to the α*CR* probe, the isoform-specific α*11* probe showed a mosaic staining pattern (Figure 4A e). Similar mosaic patterns were observed with other α isoform-specific probes (data not shown, and see α*1* in Figure 6E). The differential expression of *Pcdh*-α isoforms in single neurons is also observed in periglomerular cells (Kohmura et al., 1998) and mitral/tufted cells (data not shown) in the OB. These results suggested that individual OSNs and neurons in the OB differentially express *Pcdh*-α isoforms, similar to Purkinje cells (Esumi et al., 2005).

A previous single-cell RT-PCR analysis of Purkinje cells revealed strong evidence for the stochastic and combinatorial expression of *Pcdh*-α isoforms in individual neurons (Esumi et al., 2005). We therefore performed single-cell RT-PCR analysis of the OSNs to determine the expression pattern of Pcdh-α isoforms in these neurons at the single-cell level. Using OMP-GFP mice, we picked up GFP-positive cells. Of 30 GFP-positive OSNs, 18 were OMP positive and also had a product representing at least one of the 12 *Pcdh*-α variable isoforms (Figure 4B). Each single OSN expressed a different combination of *Pcdh*-α isoforms, except for four α*2*-positive and two α*4*-positive cells. Although this one-tube single-cell RT-PCR method has more experimental limitations than the split single-cell RT-PCR method, these results indicated that individual OSNs differentially express *Pcdh*-α isoforms, similar to Purkinje cells.

### Axonal coalescence in mutant mice in which the α*2* to α*C2* exons in the variable region of the Pcdh-α cluster are deleted

**Figure 5:**
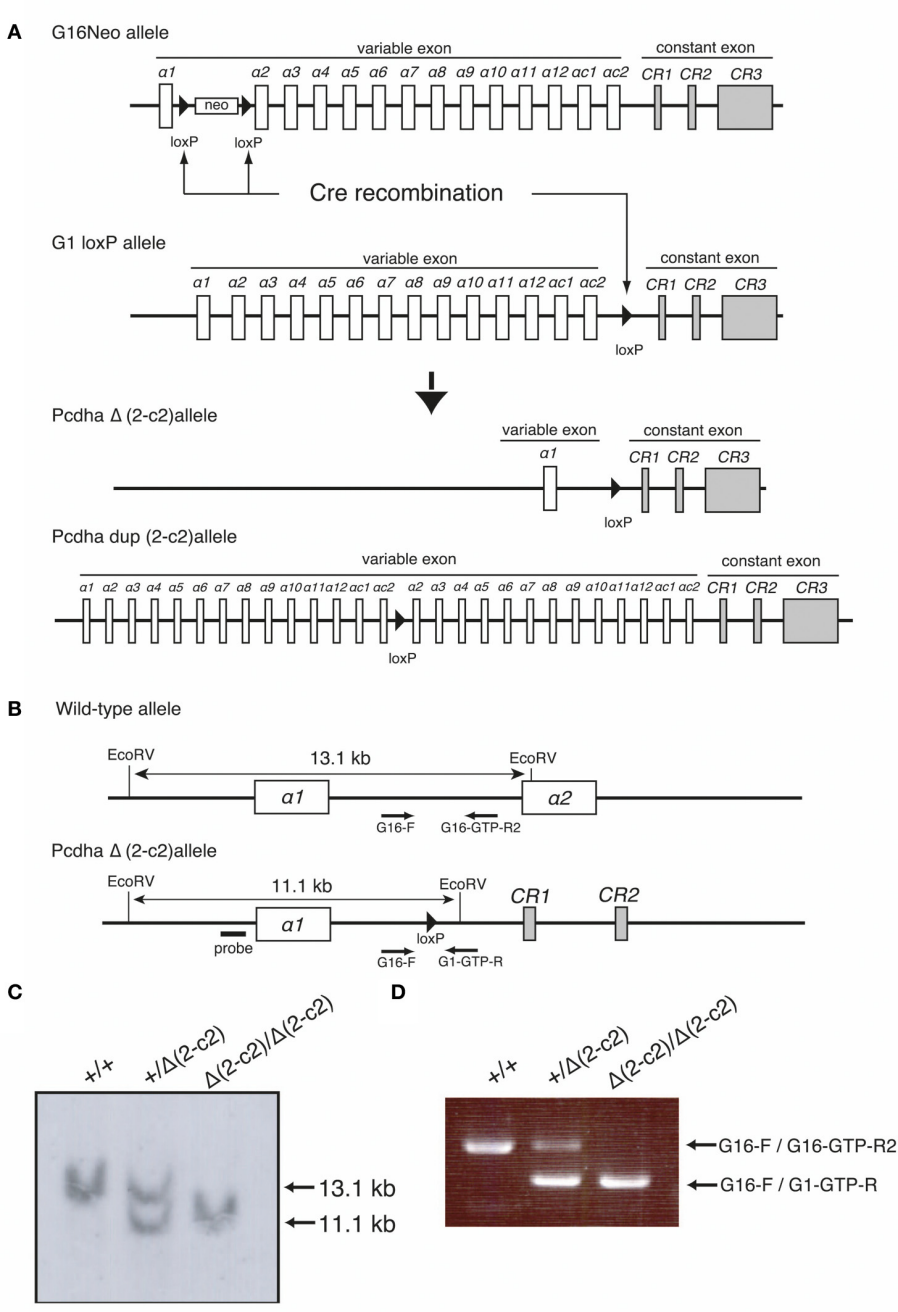
**Generation of the *Pcdha*^Δ(*2–c2*)^ and *Pcdha*^*dup*(*2–c2*)^ alleles in mice. (A)** *Pcdha*^Δ(*2–c2*)^ and *Pcdha*^*dup*(*2–c2*)^ mice were generated by mating G16Neo mice, G1 loxP mice, and Sycp-Cre transgenic mice created by synaptosomal-Cre (TAMERE) system in the testis. This Cre is expressed during meiotic crossing-over. **(B)** Partial genomic structures of the WT and *Pcdha*^Δ(*2–c2*)^ allele. **(C,D)** Genotyping of WT (+/+), *Pcdha*^+/Δ(*2–c2*)^ [+/Δ (*2–c2*)], and *Pcdha*^Δ(*2–c2*)/Δ(*2–c2*)^ [*Δ *2–c2*/Δ(*2–c2*)*] by Southern blot and PCR analyses (see “Materials and Methods”).

**Figure 6:**
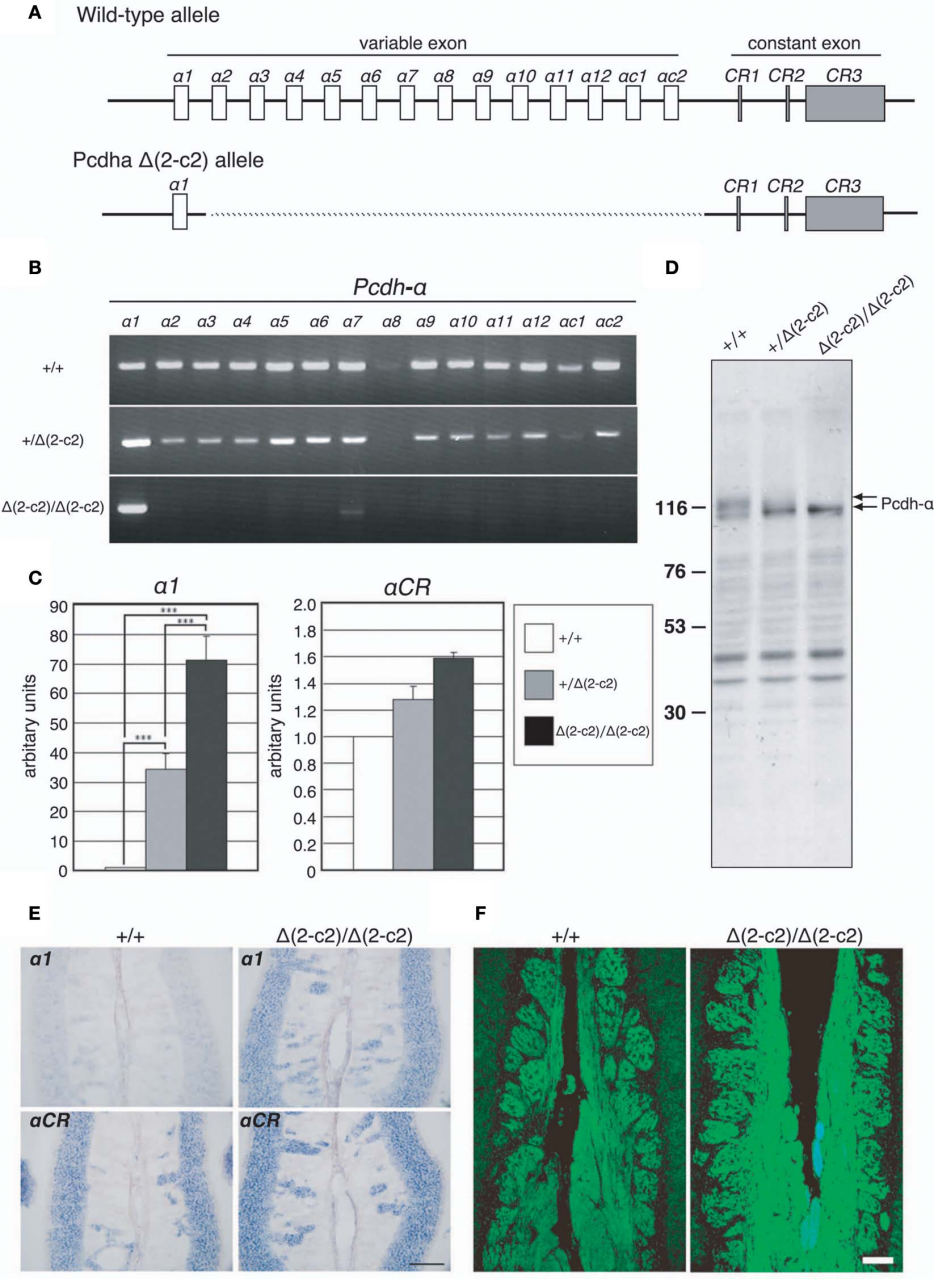
***Pcdha*^Δ(*2–c2*)/Δ(*2–c2*)^ deletion mutant mice. (A)** Wild-type *Pcdh*-α genes consist of variable-region (α*1* to α*12*, α*c1* and α*c2*) and constant-region (*CR1*–*CR3*) exons. The individual variable exons are transcribed from their own promoters. A *Pcdh*-α transcript is produced from one variable exon and three or four constant exons by splicing. In the *Pcdha*^Δ(*2–c2*)/Δ(*2–c2*)^ mice, exons α*2–αc2* were deleted, leaving only exon α*1* in the variable region. **(B)** RT-PCR analysis of brain extracts of WT (+/+), *Pcdha*^+/Δ(*2–c2*)^ [*+/Δ(*2–c2*)*], and *Pcdha*^Δ(*2–c2*)/Δ(*2–c2*)^ Δ(*2–c2*/Δ*2–c2*) mice. **(C)** qRT-PCR analysis of α*1* transcripts in the brain of WT (+/+), *Pcdha*^+/Δ(*2–c2*)^ [*+/Δ(*2–c2*)*], and *Pcdha*^Δ(*2–c2*)/Δ(*2–c2*)^ [*Δ(*2–c2*)/Δ(*2–c2*)*] mice. ^***^P < 0.0001, vs. WT and vs. *Pcdha*^+/Δ(*2–c2*)^. Data are shown as the mean ± S.D. **(D)** Immunoblotting analysis of brain lysates with an anti-Pcdhα CR antibody. **(E)** Expression of α*1* and α*CR* transcripts in OSNs of the OE were examined by *in situ* hybridization histochemistry. Constitutive expression of α*1* transcripts was seen in the OSNs of *Pcdha*^Δ(*2–c2*)/Δ(*2–c2*)^ [*Δ(*2–c2*)/Δ(*2–c2*)*] mice. Scale bar, 100 μm. **(F)** Pcdh-α immunoreactivity with an anti-Pcdhα CR antibody was strong in the OSN axons and glomeruli of both WT (+/+) and *Pcdha*^Δ(*2–c2*)/Δ(*2–c2*)^ [*Δ(*2–c2*)/Δ(*2–c2*)*] mice. Scale bar, 100 μm.

Our findings indicated that Pcdh-α proteins are essential for the homotypic axonal coalescence of OSNs; however, it was not known whether all or some specific Pcdh-α isoforms were necessary for this coalescence. To address this question, we produced and analyzed a *Pcdh*-α variable exon-deletion mutant line: *Pcdha*^Δ(*2–c2*)/Δ(*2–c2*)^ mice (Figure 5), in which the α*2*–α*c2* exons were deleted, and only α*1* remained, in the variable region of the *Pcdh*-α cluster (Figure 6A). In the *Pcdha*^Δ(*2–c2*)/Δ(*2–c2*)^ mice, the remaining α*1* isoform was highly expressed in the brain (Figure 6B). The expression level of the α*1* gene in the *Pcdha*^Δ(*2–c2*)/Δ(*2–c2*)^ mouse brain increased by approximately 70-folds compared to WT (Figure 6C), while the total level of *Pcdh*-α expression detected by the common cytoplasmic region was similar between the *Pcdha*^Δ(*2–c2*)/Δ(*2–c2*)^ and WT mice. In immunoblots of brain extract samples, broad bands were immunostained by an anti-Pcdh-α CR antibody (Murata et al., 2004) that recognized a common cytoplasmic region of Pcdh-α isoforms in WT mice; in contrast, a sharp band was strongly stained in the *Pcdha*^Δ(*2–c2*)/Δ(*2–c2*)^ mice (Figure 6D). We previously reported that in deletion mutants of the variable region of the *Pcdh*-α cluster, the missing exons are efficiently compensated for by the remaining variable exons (Noguchi et al., 2009). Therefore, to examine the compensation of *Pcdh*-α expression by the remaining α*1* gene in the OSNs of *Pcdha*^Δ(*2–c2*)/Δ(*2–c2*)^ mice, we performed *in situ* hybridization analysis of the OE of WT and *Pcdha*^Δ(*2–c2*)/Δ(*2–c2*)^ mice with α*1* and α*CR* probes (Figure 6E). Interestingly all the OSNs in the OE of *Pcdha*^Δ(*2–c2*)/Δ(*2–c2*)^ mice extensively expressed the α*1* isoform similar to that of α*CR*, while the OSNs of WT mice rarely expressed α*1*. However, the expression pattern and level of the total *Pcdh*-α isoforms detected with the α*CR* probe were not markedly changed (Figure 6E). Immunostaining with the anti-Pcdh-α CR antibody also showed that the distribution pattern and level of the Pcdh-α proteins were not markedly different between the WT and *Pcdha*^Δ(*2–c2*)/Δ(*2–c2*)^ mice (Figure 6F). In *Pcdha*^Δ(*2–c2*)/Δ(*2–c2*)^ mice, the α1 protein was extensively distributed throughout the OSN axons and glomeruli. These results indicated that the α1 isoform completely compensated for the expression of the other *Pcdh*-α isoforms in all the OSNs and neurons of the OBs.

**Figure 7:**
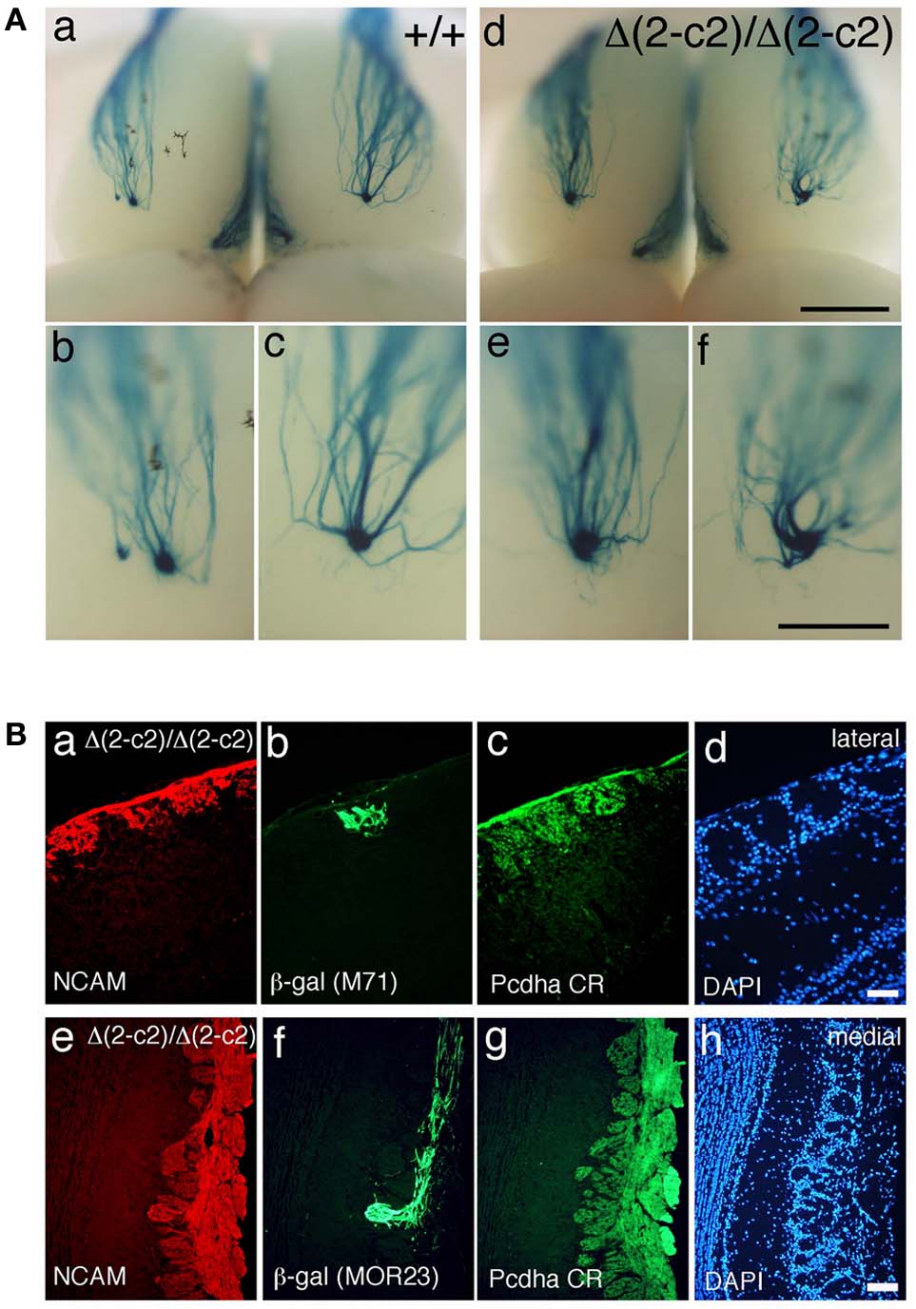
**Axonal coalescence of OSN projections in *Pcdha*^Δ^(*2–c2)/Δ(*2–c2*)* mice. (A)** X-gal-stained dorsal M71 glomeruli in WT (+/+) and *Pcdha*^Δ(*2–c2*)/Δ(*2–c2*)^ (*Δ(*2–c2*)/Δ(*2–c2*)*) mice at P30. Glomeruli appeared normal in the *Pcdha*^Δ(*2–c2*)/Δ(*2–c2*)^ mice. Scale bars, 1 mm for panels (a, d), and 500 μm for panels (b, c, e, and f). **(B)** Analysis of M71 (b) and MOR23 (f) glomeruli in the *Pcdha*^Δ(*2–c2*)/Δ(*2–c2*)^ mice. Sections of OBs were double-labeled with β-galactosidase (β-gal; green) and NCAM (red) (a, e) antibodies. Each adjacent section (c, d and g, h) was stained with anti-Pcdha CR antibody and 4′,6-diamidino-2-phenylindole (DAPI; blue). Scale bars, 50 μm for panels (a–d) and 100 μm for panels (e–h).

We next examined the axonal coalescence of homotypic OSNs of the *Pcdha*^Δ(*2–c2*)/Δ(*2–c2*)^ mice. We crossed the *Pcdha*^Δ(*2–c2*)/Δ(*2–c2*)^ mice with M71-IRES-taulacZ or MOR23-IRES-taulacZ mice. In whole-mount OB preparations of M71-IRES-taulacZ or MOR23-IRES-taulacZ mice at P30, there was typically one labeled glomerulus per half-bulb at the lateral and medial side in both WT and *Pcdha*^Δ(*2–c2*)/Δ(*2–c2*)^ mice (Table 2) (Figure 7A). Sectional analyses of the MOR23 glomeruli showed that the average number of glomeruli in both the lateral and medial sides of the half-bulbs were not significantly different between the WT and *Pcdha*^Δ(*2–c2*)/Δ(*2–c2*)^ mice (Table 2). Whole-mount analyses of the M71 glomeruli also showed no significant difference between the WT and *Pcdha*^Δ(*2–c2*)/Δ(*2–c2*)^ mice in the average number of glomeluli in both the lateral and medial sides of the half-bulbs (Table 2). The coalescence of M71 and MOR23 axons appeared normal in the *Pcdha*^Δ(*2–c2*)/Δ(*2–c2*)^ mice, in which the α1 protein was extensively enriched in all the OSN axons and glomeruli (Figure 7B). In addition, immunostaining of adjacent sections with the anti-Pcdhα CR antibody revealed that the α1 protein was distributed in both the lateral M71 and medial MOR23 glomeruli of the OB in the *Pcdha*^Δ(*2–c2*)/Δ(*2–c2*)^ mice (Figure 7B). These results indicated that a diversity of Pcdh-α isoforms in OSNs is not always required for the axonal coalescence of M71 and MOR23 homotypic OSNs into glomeruli. Instead, constitutive expression of the α1 isoform in neurons including OSNs was sufficient for the normal coalescence and elimination of OSN projections.

**Table 2:** **Number of glomeruli per half-bulb**.

	**Age**	**(*n*)**	**Lateral**	**(Min–Max)**	**(*n*)**	**Medial**	**(Min–Max)**
**50 μM–THICK SECTION**							
MOR23	P30						
WT		(12)	1.0	( – )	(12)	1.0	( – )
Δ (*2–c2*)/Δ (*2–c2*)		(16)	1.0	( – )	(16)	1.1	(1.0–2.0) *P* = 0.2120
**WHOLE-MOUNT**							
M71	P30						
WT		(14)	1.4	(1.0–3.0)	(14)	1.5	(1.0–3.0)
Δ (*2–c2*)/Δ (*2–c2*)		(16)	1.7	(1.0–3.0) *P* = 0.4620	(16)	1.1	(1.0–2.0) *P* = 0.0191
M71	P30						
WT		(10)	1.2	(1.0–2.0)	(10)	1.1	(1.0–2.0)
Δ A/Δ A		(10)	2.1	(1.0–3.0) *P* = 0.0150	(10)	1.6	(1.0–2.0) *P* = 0.0223

n = half-bulb, Mann–Whitney U-test

### Requirement of the common cytoplasmic region of Pcdh-α proteins for the normal coalescence and elimination of ectopic OSN projections in glomeruli

**Figure 8:**
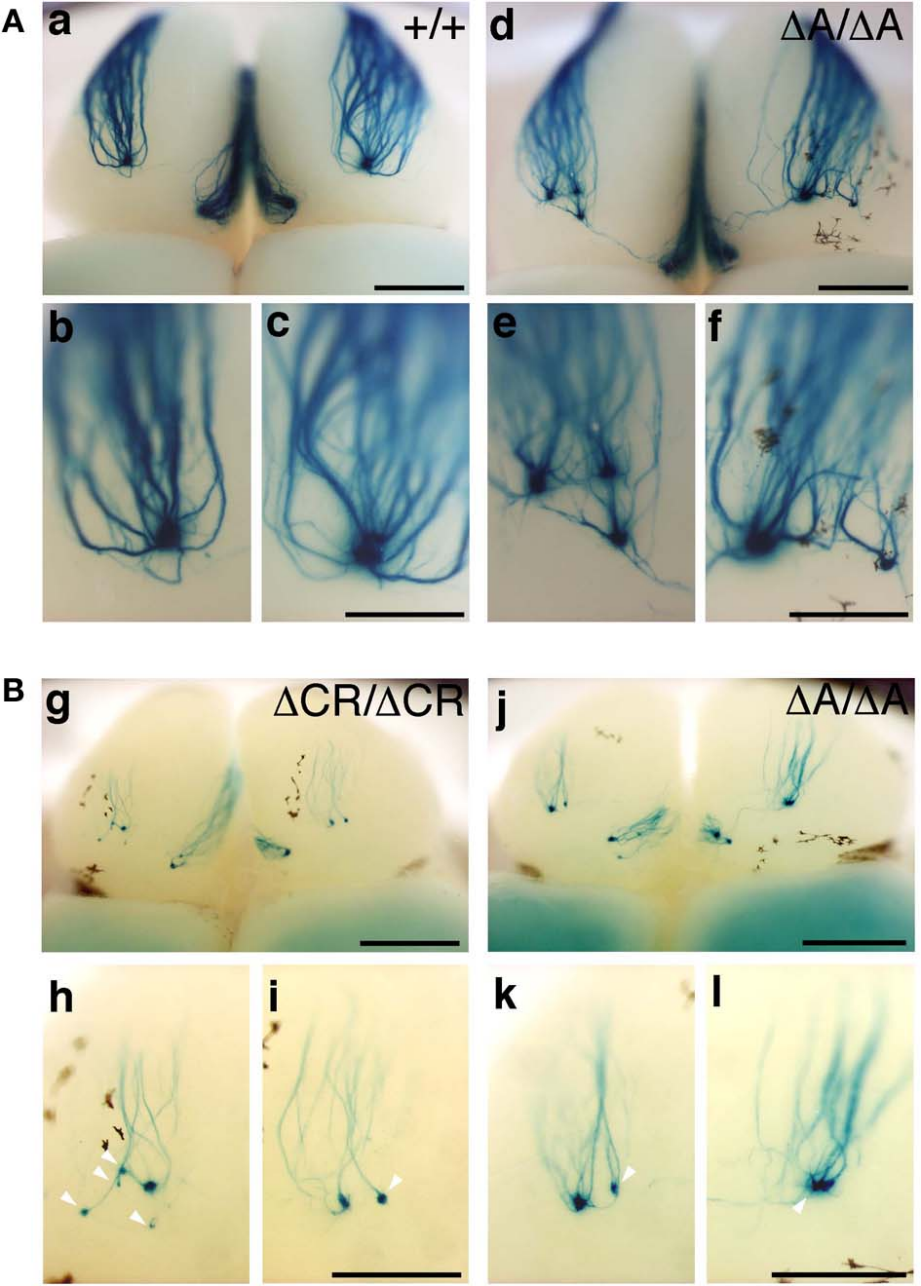
**M71 glomeruli in *Pcdha*^Δ*A*/Δ*A*^ and *Pcdha*^Δ*CR*/Δ*CR*^ mice. (A)** X-gal-stained M71 glomeruli in WT (a–c) and *Pcdha*^Δ*A*/Δ*A*^ (d–f) mice at P30. Multiple small, extraneous glomeruli were seen. **(B)** M71 glomeruli in *Pcdha*^Δ*CR*/Δ*CR*^ (g–i) and *Pcdha*^Δ*A*/Δ*A*^ (j–l) mice at P7. The abnormalities found in *Pcdha*^Δ*A*/Δ*A*^ (*ΔA/ΔA*) mice were similar to those of *Pcdha*^Δ*CR*/Δ*CR*^ (*ΔCR/ΔCR*) mice through development (P7–P60). Unlike these mice, axonal coalescence of the *Pcdha*^Δ(*2–c2*)/Δ(*2–c2*)^ mice appeared normal at least for the M71 and MOR23 OSNs (see Figure 7). Scale bars, 1 mm for panels (a) and (d), 500 μm for panels (b, c, e, f, g, and j), and 250 μm for panels (h, i, k, and l).

*Pcdha*^Δ*CR*/Δ*CR*^ mice are presumptive null mutants of the *Pcdh*-α locus; no Pcdh-α proteins are seen in the brain of these mice (Hasegawa et al., 2008). Another *Pcdh*-α mutant mouse, *Pcdha*^Δ*A*/Δ*A*^, lacks the common cytoplasmic region (56 amino acids) of Pcdh-α A-type isoforms, and expresses a truncated Pcdh-α protein that lacks the A-type specific cytoplasmic tail (Katori et al., 2009). *Pcdha*^Δ*A*/Δ*A*^ mice show disrupted and diffuse serotonergic axon projections, similar to those of the *Pcdha*^Δ*CR*/Δ*CR*^ mice (Katori et al., 2009). To further address how the Pcdh-α proteins control the axonal coalescence of homotypic OSNs expressing specific ORs, we analyzed the axonal coalescence in *Pcdha*^Δ*A*/Δ*A*^ mice with the M71-IRES-taulacZ locus. We found a perturbed coalescence of M71 axons in the *Pcdha*^Δ*A*/Δ*A*^ mice at P30 (Figure 8A) and P7 (Figure 8B). Similar abnormalities were found in the *Pcdha*^Δ*CR*/Δ*CR*^ mice (Figure 8B, Hasegawa et al., 2008). Whole-mount analysis of the M71-IRES-taulacZ mice typically showed one labeled glomerulus per half-bulb at the lateral and medial side and in rare cases a second glomerulus; thus, there was an average of 1.1–1.2 glomeruli per lateral or medial side of the half-bulb at P30 in WT mice. In contrast, *Pcdha*^Δ*A*/Δ*A*^ mice showed a perturbed coalescence of M71 axons and significantly higher numbers of M71 glomeruli compared to WT mice (Table 2). These results indicated that the common cytoplasmic region among Pcdh-α proteins is required for both the initial coalescence of OSN axons and the elimination of glomeruli during development.

### No effect of the neural activity of OSNs on the expression level and distribution pattern of Pcdh-α

**Figure 9:**
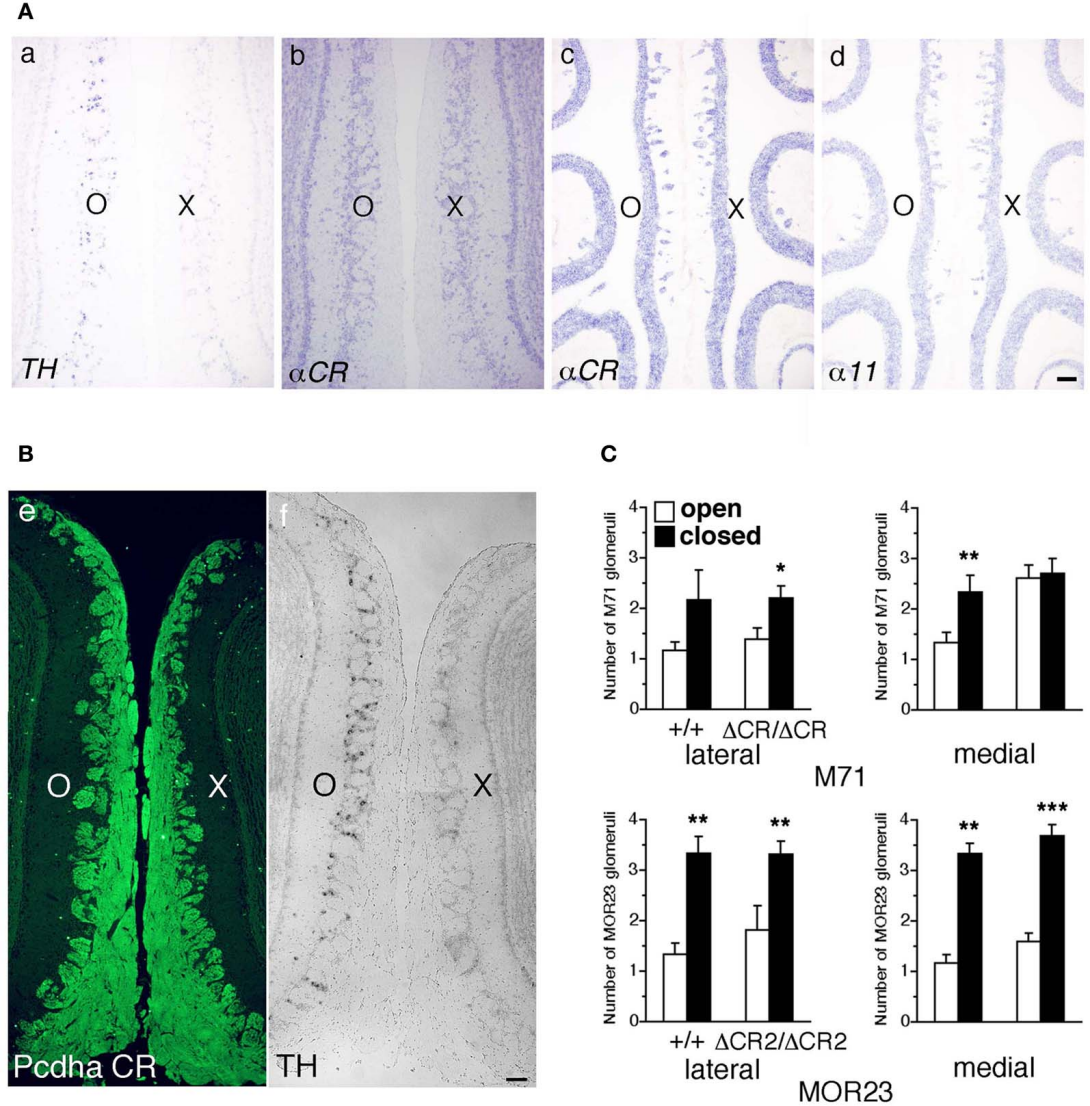
**Expression of *Pcdh*-α and the number of M71 and MOR23 glomeruli after naris occlusion of WT and *Pcdh*-α-deficient (*Pcdha*^Δ*CR*/Δ*CR*^ and *Pcdha*^Δ*CR*2/Δ*CR*2^) mice. (A)** A WT mouse was subjected to unilateral naris closure at P21 and analyzed after 1 week. The expression of tyrosine hydroxylase (*TH*) (a) and α*CR* transcripts (b) of the closed (X) and open (O) sides in the OB were examined by *in situ* hybridization histochemistry. The expression of α*11* and α*CR* transcripts in the OE were also examined (c, d). Scale bar, 100 μm. **(B)** A WT mouse was subjected to naris occlusion at P5 and analyzed at P30. Pcdh-α immunoreactivity with the anti-Pcdhα CR antibody was strong in OSN axons and glomeruli in both the closed (X) and open (O) sides at similar levels (e), in contrast to the change in TH signals (f). Scale bar, 100 μm. **(C)** The number of M71 and MOR23 glomeruli per lateral and medial half-bulb of the open (white bars) and closed (black bars) sides after naris occlusion in WT (+/+) and *Pcdh*-α-deficient (*Pcdha*^Δ*CR*/Δ*CR*^ and *Pcdha*^Δ*CR*2/Δ*CR*2^) mice at P30. Although ectopic glomeruli were further increased in *Pcdha*^Δ*CR*/Δ*CR*^ (*ΔCR/ΔCR*) and *Pcdha*^Δ*CR*2/Δ*CR*2^ (*ΔCR2/ΔCR2*) mice by the treatment, the total number of glomeruli after treatment was almost the same in the WT and *Pcdh*-α-deficient (*Pcdha*^Δ*CR*/Δ*CR*^ and *Pcdha*^Δ*CR*2/Δ*CR*2^) mice. The number of MOR23 glomeruli in *Pcdha*^Δ*CR*2/Δ*CR*2^ mice are shown in Table 1. Significant differences at ^*^P < 0.05, ^**^P < 0.01, and ^***^P < 0.001 calculated by the Mann–Whitney *U*-test.

Axon guidance molecules showing OR-specific expression are often regulated by the neural activity of OSNs (Serizawa et al., 2006; Kaneko-Goto et al., 2008); therefore, we examined whether a reduction in OSN neural activity would affect the Pcdh-α expression. WT mice were subjected to unilateral naris occlusion at 3 weeks and analyzed by *in situ* hybridization histochemistry after 1 week. Efficiency of the naris occlusion was validated by the loss of tyrosine hydroxylase (TH) signals from the glomerular layer on the closed side of the OB (Figure 9A a) (Stone et al., 1990). In contrast, mRNA signals of the isoform common α*CR* probe in the OE were quite similar between the open and closed sides (Figure 9A b). In the OE, the expression levels with the α*11* and α*CR* probes were quite similar between the open and closed sides (Figure 9A c, d). Furthermore, immunohistochemistry of the P30 WT mouse after naris occlusion showed that the distribution and intensity of Pcdh-α immunoreactivity in the OB were almost the same between the closed and open sides (Figure 9B). These results indicated that the Pcdh-α expression in the OSNs and OB was not altered by the OSN neural activity.

**Figure 10:**
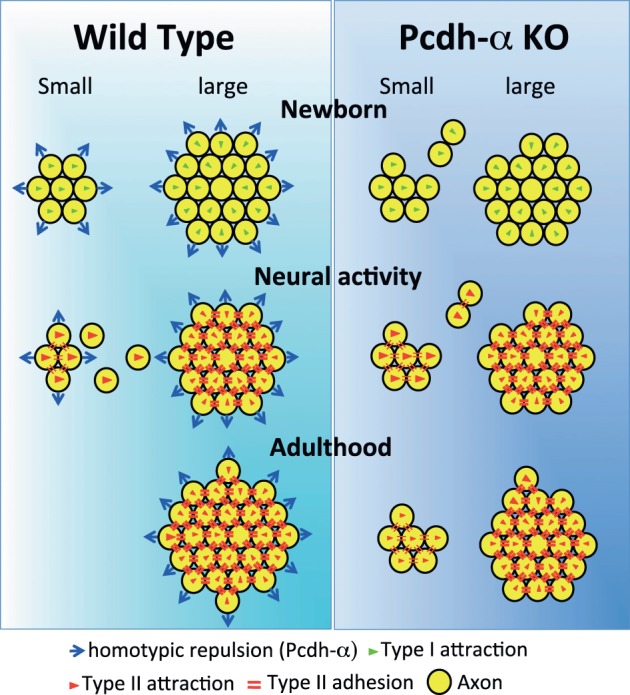
**Possible mechanism for the coalescence and elimination of homotypic OSN projections mediated by Pcdh-α proteins.** In newborn mice, homotypic OSN axons coalesce by Type I attraction (green triangles). Type I attraction is performed by axon-target interaction by guidance molecules in a graded manner, e.g., Sama3A/Neuropilin1. Pcdh-α protein may elicit a contact-induced repulsion between homotypic OSN axons. In *Pcdh*-α-deficient mice, small glomeruli were generated because the contact-induced repulsive activity did not occur. During development, neural activity promotes Type II attraction (red triangles) and the Type II adhesion (red double-bars) of homotypic OSN projections. Type II attraction depends on axon-axon interactions during contact-induced repulsion between heterotypic OSN axons, e.g., mediated by EphA5/ephrin-A5. Type II adhesion results from the induction of expression of homophilic cell adhesion molecules, e.g., Kirrel2/Kirrel3. In small ectopic glomeruli, these attractive functions promote the correct coalescence of homotypic OSN projections and the elimination of ectopic projections. Pcdh-α protein may help to eliminate small ectopic glomeruli by the contact-induced repulsion of homotypic OSN axons. In adulthood, multiple small ectopic glomeruli disappear in WT mice, but are maintained in *Pcdh*-α-deficient mice. The interdependence of OSN coalescence could result from the balance between the neural-activity-induced attraction by guidance molecules and the contact-induced repulsion by Pcdh-α proteins in the glomerulus.

Naris occlusion leads to sensory deprivation, which might inhibit the neural activity-regulated process by which multiple glomeruli are eliminated. Indeed, a previous study showed that this treatment significantly increases the number of glomeluli (Zou et al., 2004). Therefore, we next analyzed the number of M71 and MOR23 glomeruli between the closed and open sides of the OB to examine the effects of naris occlusion in the *Pcdha*^Δ*CR*/Δ*CR*^ or *Pcdha*^Δ*CR*2/Δ*CR*2^ mice. Although the number of ectopic glomeruli was already significantly increased in these *Pcdh*-α-deficient mice, unilateral naris occlusion further increased the number of M71 and MOR23 glomeruli in the WT and *Pcdh*-α-deficient (*Pcdha*^Δ*CR*/Δ*CR*^ and *Pcdha*^Δ*CR*2/Δ*CR*2^) mice until they all reached similar levels (Figure 9C). Thus, even in *Pcdh*-α-deficient mice, the ectopic glomeruli were further increased by reduced neural activity. Together, these findings further suggest that Pcdh-α proteins function continuously to organize the projections of OSN axons and eliminate ectopic glomeruli in a neural activity-independent manner (Figure 10).

## Discussion

We previously reported that loss of the Pcdh-α family (*Pcdha*^Δ*CR*/Δ*CR*^) disrupts the coalescence of OSN axons into glomeruli (Hasegawa et al., 2008). However, the relationship between the diverse Pcdh-α isoforms and axonal coalescence was not well understood. In the present study, we found that Pcdh-α isoform expression and function in the olfactory system are different from those of other known olfactory guidance molecules. First, the phenotypes of axonal coalescence of OSNs in *Pcdh*-α-deficient mice were not restricted to specific OR-expressing OSNs but rather were found in all kinds of homotypic OSNs. Second, the expression of Pcdh-α molecules was not altered by the neural activity of OSNs. Third, following *Pcdh*-α deficiency, the numbers of ectopic glomeruli were even further increased by a reduction in neural activity, suggesting that Pcdh-α's functions in coalescing and eliminating abnormal OSN axons are not dependent on neural activity. Fourth, the constitutive expression of Pcdh-α protein in neurons including OSNs was essential for normal axonal coalescence, although the diversity of Pcdh-α proteins was not always needed. In addition, we found that a common cytoplasmic region among Pcdh-α proteins was essential for the axonal coalescence and elimination of ectopic glomeruli. Taken together, we conclude that the Pcdh-α family is constitutively involved in mechanisms regulating the coalescence of OSN axons and elimination of small ectopic glomeruli that are independent of OR-specificity and neural activity.

Ebrahimi and Chess (2000) proposed a mechanism for maintaining the axonal coalescence of OSNs, in which the presence of other OSNs expressing the same OR is required, called “interdependence.” The projections of OSNs expressing an ectopic OR always coalesce in newborn mice, although this coalescence is not always maintained in adult mice. The probability of maintaining coalescence in adult mice is positively correlated with the number of OSNs expressing the OR (Ebrahimi and Chess, 2000). Thus, there must be some mechanism for removing the excess glomeruli. In the *Pcdh*-α-mutant mice, even small glomeruli do not disappear in adulthood. The present data showed that the constitutive expression and cytoplasmic region of Pcdh-α proteins are required for the elimination of miswired axons.

### Pcdh-α molecules may function in a neural activity-independent manner

It has been suggested that neural activity is required for the establishment and maintenance of specific glomeruli (Zou et al., 2004). Blocking the neural activity in OSNs by overexpression of the inward rectifying potassium channel (Kir2.1) induce multiple abnormal glomeruli in P2, MOR28 and MOR23 axons (Yu et al., 2004). On the other hand, in olfactory cyclic nucleotide-gated channel subunit 1 (OCNC1)-deficient mice, M72 axons form multiple abnormal glomeruli, but P2 axons do not (Lin et al., 2000; Zheng et al., 2000). Thus, P2 axons are probably less affected by a reduction in neural activity than other axon types in determining their targeted projections to the OB.

Interestingly, the *Pcdh*-α-deficient mice in our present study clearly showed abnormal multiple glomeruli in all OR-expressing OSNs examined, even in the P2 axons. Although it was already known that the elimination of ectopic glomeruli proceeds through the neural activity of OSNs, our present data suggest that Pcdh-α proteins may function in glomerular remodeling in a neural activity-independent fashion.

### Role of the cytoplasmic region of Pcdh-α proteins

The abnormalities in the axonal coalescence of OSNs in *Pcdha*^Δ*A*/Δ*A*^ mice were similar to those of *Pcdha*^Δ*CR*/Δ*CR*^ mice, indicating that the cytoplasmic region of the Pcdh-α protein is essential for the axonal coalescence of homotypic OSNs. The cytoplasmic domain of Pcdh-α is known to bind cytoplasmic signaling proteins of the focal adhesion kinase (FAK) family, FAK and PYK2 (Chen et al., 2009). The FAK family contributes to signaling cascades that regulate growth cones (Chacon and Fazzari, 2011) and membrane stabilization, with PKC and MARCKS (Garrett et al., 2012). Interestingly, MARCKS-like and GAP43 proteins down-stream of PKC are extensively expressed in immature OSNs (McIntyre et al., 2010). Although a role for FAK-PKC signaling has not been reported in OSN axons, it may be involved in regulating coalescence and the elimination of glomeruli.

### Loss of Pcdh-α molecular diversity: deletion of exons α*2* to α*C2* in the variable region of the P*cdh*-α cluster

In the *Pcdh*-α cluster, the variable region encodes multiple first exons (variable exons) for 14 different *Pcdh*-α isoforms. Each variable exon is transcribed from its own promoter and *cis*-spliced to the constant region exons, which are common to all the *Pcdh*-α isoforms. While the α*1*–α*12* isoforms are expressed randomly, isoforms α*c1*–α*c2* are expressesd constitutively in individual Purkinje cells (Esumi et al., 2005; Kaneko et al., 2006). Here we showed that *Pcdh*-α isoforms were also constitutively expressed in the OSNs of the OE, in which the α*1*–α*12* isoforms were randomly expressed, indicating that the mechanism of *Pcdh*-α gene regulation is similar in Purkinje cells and OSNs. Interestingly, in *Pcdha*^Δ(*2–c2*)/Δ(*2–c2*)^ mice, in which exons α*2*–α*c2* in the variable region of the *Pcdh*-α cluster were deleted, the remaining α*1* isoform compensated for the others, and was expressed constitutively in neurons including OSNs (Figure 6). Such compensation of missing *Pcdh*-α cluster genes is also found in other deletion mutants, *Pcdha*^Δ(11−*c*2)/Δ(11−*c*2)^ and *Pcdha*^Δ(2−11)/Δ(2−11)^ (Noguchi et al., 2009). In these mice, the total expression level of *Pcdh*-α isoforms is maintained, and the remaining isoforms compensate for those missing. This compensation can be explained by a mechanism in which a *cis*-element for regulating the *Pcdh*-α cluster selects one or two gene(s) by binding within the α*1*–α*12* promoters. In fact, the *cis*-element for the *Pcdh*-α cluster was identified as HS5-1 (Ribich et al., 2006), and shown to be regulated in Purkinje cells *in vivo* (Yokota et al., 2011; Monahan et al., 2012). In addition, a chromatin factor known as CCCTC-binding factor (CTCF) is essential for gene regulation of the *Pcdh*-α cluster (Hirayama et al., 2012). Here, in the *Pcdha*^Δ(*2–c2*)/Δ(*2–c2*)^ mice, the same *Pcdh*-α gene-regulation mechanisms might function in individual OSNs of the OE and in neurons of the OBs. In any case, the *Pcdha*^Δ(*2–c2*)/Δ(*2–c2*)^ mice had normal-looking glomeruli in the OB, indicating that the constitutive expression, but not the diversity, of Pcdh-α protein is essential for the axonal coalescence of OSNs and the elimination of small ectopic glomeruli in the olfactory system.

### Possible mechanism for the involvement of Pcdh-α protein in the coalescence and elimination of homotypic OSN axons

The development of OSN axon projections and their coalescence into glomeruli from the OE to the OB involves several molecular mechanisms regulated by step-wise processes. The stepwise regulation of OSN projection is categorized into type I for immature OSNs and type II for mature OSNs (Sakano, 2010). Immature and mature OSNs that express the same OR possess similar levels of the same guidance cues, and these homotypic axons project and coalesce to the same glomerulus position in the OB (Sakano, 2010).

Pcdh-α proteins may constitutively function to determine the organization of the OSN axon projections. We propose that the *Pcdh*-α family uses a novel mechanism for axonal coalescence and the elimination of ectopic glomeruli for all OR-expressing homotypic OSNs. The coalescence activity enhances the specific topography and OR-signaling-dependent neural activity mediated by guidance molecules (Sakano, 2010). Previously identified olfactory guidance molecules are known to contribute exclusively to the coalescence of homotypic OSN axons into rudimentary glomeruli, in a manner regulated by distinct levels of homophilic and repulsive activities of the OR-specific OSNs. However, no guidance molecules providing the homotypic repulsion for axonal elimination had been identified in the olfactory system. Here we propose that Pcdh-α proteins provide a repulsive activity for all homotypic OSNs and have a counterbalancing effect on the axonal coalescence mechanisms mediated by previously known olfactory guidance molecules (Figure 10). This Pcdh-α-mediated signaling provides a novel mechanism for eliminating OR-expressing OSN axons, that is, a repulsive signaling arising from all types of glomeruli that are independent of topography or neural activity. Consequently, this hypothesis raises the further possibility that Pcdh-α could be a determinant of the specification of a major (large) glomerulus from other minor (small) glomeruli of homotypic OSNs. Pcdh-α protein is extensively expressed in both the presynaptic side of OSNs and the postsynaptic side of mitral/tufted and periglomerular cells, suggesting it may function in the appropriate maintenance and elimination of synaptic connections in homotypic glomeruli. Therefore, we propose a possible mechanism in which the interdependence of homotypic OSNs in axonal coalescence is ensured by a balance between the neural-activity-induced attraction by many guidance molecules and the contact-induced repulsion by Pcdh-α proteins in the glomeruli of the OB (Figure 10).

### Conflict of interest statement

The authors declare that the research was conducted in the absence of any commercial or financial relationships that could be construed as a potential conflict of interest.
